# Human physiological and metabolic responses to an attempted winter crossing of Antarctica: the effects of prolonged hypobaric hypoxia.

**DOI:** 10.14814/phy2.13613

**Published:** 2018-03-09

**Authors:** Katie A. O'Brien, Ross D. Pollock, Mike Stroud, Rob J. Lambert, Alex Kumar, Robert A. Atkinson, David A. Green, Ana Anton‐Solanas, Lindsay M. Edwards, Steve D. R. Harridge

**Affiliations:** ^1^ Centre of Human and Aerospace Physiological Sciences King's College London London United Kingdom; ^2^ Department of Physiology, Development and Neuroscience University of Cambridge Cambridge United Kingdom; ^3^ Biomedical Research Centre in Nutrition Southampton University Hospitals Trust Southampton United Kingdom; ^4^ Department of Trauma and Orthopaedics Royal Infirmary of Edinburgh Edinburgh United Kingdom; ^5^ Department of Medicine and Physiology Fribourg Switzerland; ^6^ Department of Primary Care & Public Health Sciences King's College London London United Kingdom; ^7^ Centre for Biomolecular Spectroscopy and Randall Division of Cell and Molecular Biophysics King's College London Guy's Campus London London United Kingdom; ^8^ KBRwyle European Astronaut Centre European Space Agency Linder Höhe Cologne Germany; ^9^ GlaxoSmithKline Human Performance Lab Brentford United Kingdom; ^10^ Respiratory Data Sciences Group Respiratory TAU GlaxosmithKline Medicines Research Stevenage United Kingdom

**Keywords:** Acclimatization, chronic exposure, hypobaric hypoxia

## Abstract

An insufficient supply of oxygen to the tissues (hypoxia), as is experienced upon high‐altitude exposure, elicits physiological acclimatization mechanisms alongside metabolic remodeling. Details of the integrative adaptive processes in response to chronic hypobaric hypoxic exposure remain to be sufficiently investigated. In this small applied field study, subjects (*n *= 5, male, age 28–54 years) undertook a 40 week Antarctica expedition in the winter months, which included 24 weeks residing above 2500 m. Measurements taken pre‐ and postexpedition revealed alterations to glucose and fatty acid resonances within the serum metabolic profile, a 7.8 (±3.6)% increase in respiratory exchange ratio measured during incremental exercise (area under curve, *P *> 0.01, mean ± SD) and a 2.1(±0.8) % decrease in fat tissue (*P* < 0.05) postexpedition. This was accompanied by an 11.6 (±1.9) % increase (*P* > 0.001) in *V*O_2_ max corrected to % lean mass postexpedition. In addition, spine bone mineral density and lung function measures were identified as novel parameters of interest. This study provides, an in‐depth characterization of the responses to chronic hypobaric hypoxic exposure in one of the most hostile environments on Earth.

## Introduction

At high altitudes, the inspired partial pressure of oxygen (O_2_) is decreased as barometric pressure (*P*_B_) falls, thus leading to hypobaric hypoxia. Survival in these conditions requires the combination of a robust physiological response alongside metabolic remodeling to ensure adenosine triphosphate (ATP) demand is met. Studies conducting in‐depth investigation into physiological remodeling responses to chronic hypoxia are lacking. Indeed, the very definition of “chronic exposure” is unclear, with some defining it as ≥42 days (Horscroft and Murray 2014), some ≥3 months (Woolcott et al. 2015) and others as little as 2 weeks (Siebenmann et al. 2016).

Irrespective of the definition, evidence suggests metabolic responses to chronic hypoxia may differ from those apparent in acute exposure. For instance, an increase in blood lactate is observed upon acute hypoxic exposure both at rest (Siervo et al. 2014) and in response to submaximal exercise (Sutton et al. 1988), a response likely linked to hypoxic inducible factor‐1α (HIF‐1α)‐dependent upregulation of glycolytic metabolism (Semenza et al. 1994; Behrooz and Ismail‐Beigi 1999; Semenza 1999; López‐Barneo et al. 2001). However, in altitude acclimatized individuals or high‐land natives, decreasing lactate accumulation in response to exercise has been reported (Hochachka et al. 2002), suggesting metabolic shifts may occur with chronic compared to acute exposure (the lactate paradox). This is alongside alterations to the mitochondrial network, with exposure to ≥42 days being associated with decreasing mitochondrial density in skeletal muscle (Horscroft and Murray 2014). Further investigation into the effects of chronic hypobaric hypoxia is therefore warranted.

It is generally considered that the decreased P_I_O_2_ experienced at altitude is the main cause of the physiological acclimatization processes. However, high‐altitude exposure usually coincides with cold ambient temperatures with the interaction between hypoxia and cold often being overlooked. This is especially pertinent when considering the metabolic effects of cold exposure.

Acclimatization to extreme cold undoubtedly relies heavily upon behavioral adaptations such as the wearing of warm clothing, but this can be accompanied by significant physiological changes that in turn influence substrate utilization, specifically increasing reliance upon carbohydrate and fat oxidation (Vallerand and Jacobs 1989).

The combination of cold exposure and hypoxia appears to modify responses to both conditions. While cold exposure is reported to increase the rate of O_2_ consumption (Vallerand and Jacobs 1989), this is attenuated at altitude (3350 m and 4360 m), a response reported to be sustained over a 6 week period, only recovering upon descent (Blatteis and Lutherer 1976). Reliance upon on shivering thermogenesis for heat generation has also been reported to increase both in acute and chronic hypoxic exposure (Blatteis and Lutherer 1976; Robinson and Haymes 1990).

In this study, the focus is upon the effects of chronic hypobaric hypoxic exposure, with subjects being exposed to high altitudes (≤2500 m) for a 24 week period during a 33 week attempted winter crossing of Antarctica. Given that temperatures on the high plateau regularly fall below −60°C, exposure to extreme cold was an inevitable factor. However, as subjects had access to living cabins and extensive protective clothing for the duration of the expedition, cold exposure was intermittent and limited to peripheries. Assessment of anthropometric and functional physiological parameters were combined with serum metabolomics to provide a broad characterization of the responses to one of the world's most hostile environments.

## Methods

### Ethical approval

Prior to measurements being made, written informed consent was obtained from all subjects. All procedures were approved by the National Health Service Wandsworth Research Ethics Committee (reference number: 12/LO/0457) and conformed to the Declaration of Helsinki.

### Expedition overview and aims

This expedition was the first ever attempted winter crossing of Antarctica. The 5 male subjects (aged 28–54 years, BMI preexpedition 26.36 ± 3.87 kg/m^2^) were assessed in the UK prior to and following their 40 week stay in Antarctica, with the attempted winter crossing lasting 33 weeks, including 24 weeks at or above 2500 m. Due to safety concerns, the crossing was halted on week 8, with subjects subsequently forced to set up winter camp, given difficulties in evacuation procedures in the winter months. Living cabins were taken along with the expedition on caterpillar trucks. Once winter camp was established, subjects spent the majority of their time residing within the cabins, only being exposed to the outside elements intermittently and whilst wearing insulating clothing.

Subjects recorded food diaries and body weight data throughout the expedition. An array of measurements were also made in the UK pre‐ and postexpedition including: assessment of metabolic profile, body composition, exercise, and lung function testing.

### Study design

**Figure 1:**
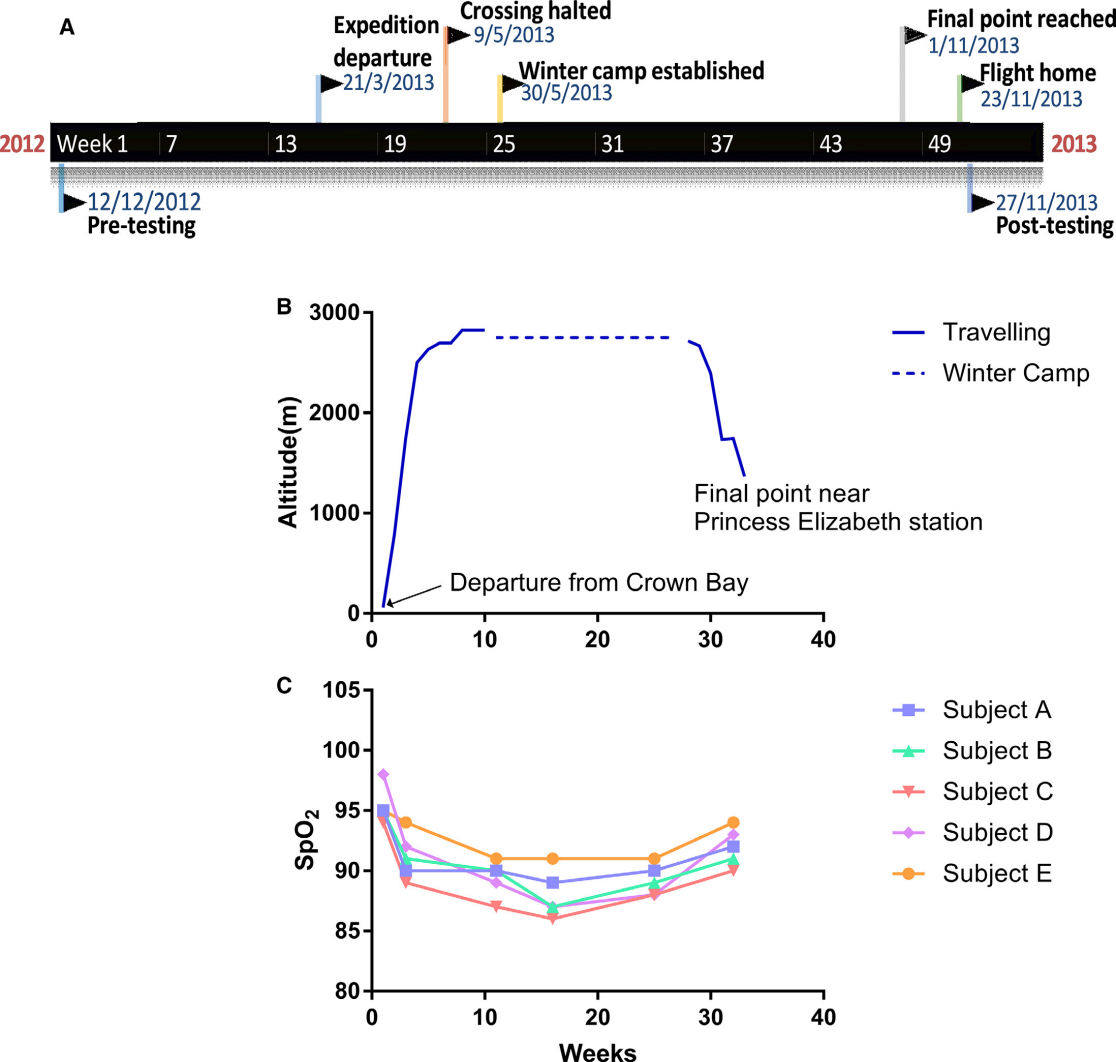
A timeline of testing and expedition details (A). A diagrammatic representation of altitude exposure during the attempted Antarctic winter crossing expedition (B) alongside corresponding SpO_2_ recorded during a moderate intensity step test (C). The 33 week attempted crossing was measured from the point at which subjects departed from Crown Bay, Antarctica. SpO_2_ was measured at intermittent points during the expedition following an 18 min moderate intensity exercise step test.

A timeline of expedition and testing details are outlined in Figure 1A, with details on altitude exposure being outlined in Figure 1B. Pretesting was conducted at King's College London on 12th December 2012, after which subjects travelled to Antarctica arriving on January 22nd. The attempted winter crossing expedition began with departure from Crown Bay on 21st March and lasted 33 weeks. During this time, subjects spent weeks 5–29 above 2500 m and reached the highest altitude of 2824 m on week 8. The crossing was halted on the 9th May and winter camp established on 30th May (week 11) at 2752 m. Subjects remained here until week 28, after which they began descending. The final point near Belgian Princess Elizabeth Station (1367 m) was reached on week 32, being 1st November 2013. Subjects remained here until 23rd November, at which point they flew home. Posttesting was conducted at King's on 27th November, 2013.

### Measurements taken during the expedition

#### Dietary intake and activity

Water (mL/day) and food intake were recorded during the expedition, the latter as total energy intake (kcal/day), broken down into contributions from: protein, fat and carbohydrate (g/day). These values were obtained from weighed food diaries in which the weight of all consumed food and water was input. Diaries were taken across a 7‐day period at 17 points within the attempted crossing time (33 weeks). Body weights, following an overnight fast and voiding of urine, were also recorded at these time points using digital balance scales.

Outside activity data were obtained from diary records taken across a 7‐day period at 8 time points within the attempted crossing time. A subjective scale was used to estimate duration of light and heavy work undertaken during these periods. Light work was roughly defined as walking or general camp maintenance and heavy work as, for instance, shoveling snow. Given the degree of subjectivity and lack of accuracy, this measure was merely used as a broad estimation and general overview of subject activity levels.

Both food and activity data are presented as an average over the 7‐day periods to present intake or activity on a “typical” day.

#### Peripheral capillary oxygen saturation (SpO_2_)

SpO_2_ was measured during a moderate exercise step test performed intermittently throughout the expedition. This 18 min test was performed using a 21.5 cm step with one step per beat of a metronome set to progressive beating rates, ending in 2 min at 160 beats per minute. SpO_2_ and pulse were measured from the index finger at the end of this test.

### Measurements taken pre‐ and postexpedition

#### Body composition

Subject height and mass were determined using a stadiometer and calibrated balance beam scales, respectively. Bone mineral density (BMD) of the whole body and lumbar spine was assessed by dual‐energy X‐ray absorptiometry (DXA) using a Hologic Discovery A scanner (Hologic, Bedford, MA). Fat mass and fat free mass were also determined from DXA scans.

#### Resting cardiovascular function

Upon arrival at the laboratory resting cardiovascular function was assessed using a Finometer Pro (Finapres Medical Systems, Amsterdam, Netherlands) with a finger cuff attached to the middle finger of the right arm. Systolic (SBP), diastolic (DBP), mean arterial pressure (MAP), and heart rate were recorded once every minute for 5 min at the end of a period of 15 min of quiet supine resting. Average values of each variable were calculated.

#### Lung function

The greatest peak expiratory flow (PEF), forced vital capacity (FVC), forced expiratory volume in 1 sec (FEV_1_) and FEV_1_/FVC% were recorded using an Oxycon Pro (CareFusion, Basingstoke, UK) calibrated prior to use. Subjects were instructed to inhale rapidly to total lung capacity and then without pausing exhale as forcefully as possible until no more air could be expired. Subjects were seated and required to maintain an upright posture during all maneuvers which were performed in accordance with the ATS/ERS Guidelines (Miller et al., 2005).

#### Serum samples

*S*erum samples were taken from fasted subjects at rest (in the morning, prior to the exercise protocol) pre and postexpedition. They were obtained by venesection in serum separating tubes and immediately spun for 10 min at 520*g* at 4^°^C. The resulting supernatant was pipetted into 2 mL polypropylene cryotubes, and immediately frozen at −80^°^C.

#### Proton nuclear magnetic resonance spectroscopy (^1^H‐NMR) for serum metabolomics

For analysis, serum samples were defrosted at room temperature and centrifuged at 16,000*g* for 10 min. A 300 *μ*L aliquot of the resulting supernatant was then mixed with 300 *μ*L of NMR buffer (250 *μ*L DSS plus phosphate buffered saline (PBS) at a concentration of 5 mmol/L added to 50 *μ*L 99.9% D_2_O). The final solution contained 8 % D_2_O for the magnetic lock. The resulting mixture was transferred to 5 mm NMR tubes within a 96‐tube rack ready for spectral acquisition, which was conducted using a Bruker Avance III 700 MHz spectrometer (Bruker Biospin, Karlsruhe, Germany) as described previously (Curtis et al. 2015).

## ^1^H‐NMR data processing and analysis

Data were processed using serial processing in Topspin (Bruker Topspin Software), applying an exponential window function with a line broadening of 0.3 Hz in the frequency domain prior to Fourier transformation. Each spectrum was subsequently phase corrected and aligned, with chemical shifts being manually referenced to DSS (at *δ* = 0 ppm), in ACD labs (ACD Labs Software.Ink), before being imported into Matlab (Mathworks, Natick, MA) at full resolution. Following this, spectra were normalized using probabilistic‐quotient normalization (Dieterle et al. 2006) and binned using adaptive intelligent binning (De Meyer et al. 2008), both in Matlab. Principal component analysis (PCA) was conducted and identification of the metabolites associated with the peaks undergoing significant changes was undertaken using Chenomx software (Chenomx NMR Suite 7.1).

#### Cardio pulmonary exercise testing

Maximal oxygen uptake ($\dot{V}O_{2}\max$) was determined using a continuous exercise test on a cycle ergometer (Lode Coriva, Lode, Groningen, Netherlands). Breath‐by‐breath measurements of O_2_ and CO_2_ concentrations as well as volume of expired air were recorded continually throughout the test (Oxycon Pro; CareFusion). Subjects wore a face mask to which the volume and gas sensors were attached. The O_2_ and CO_2_ analyzers were calibrated on each testing day with known gases in accordance with the manufacturers' guidelines. A 3 L syringe was used to calibrate the volume sensor before testing commenced.

Subjects initially cycled at a work rate of 50 W for 3 mins after which the power output continually increased until the subject could no longer continue despite strong verbal encouragement. The rate of increase in power output (1–2 W every 3–5 sec) was estimated for each subject such that maximal effort would be reached within 10–12 min. The subjects cycled at constant self‐selected rate typically between 75 and 80 rpm. Heart rate was continually monitored throughout the test using a 12 lead ECG. *V*O_2_max was determined as the greatest O_2_ uptake recorded over a 20 sec period at the end of the test. To ensure a valid $\dot{V}O_{2}\max$ was attained subjects had to meet at least two of the following criteria: (1) achievement of maximum heart rate greater than age predicted maximum (220–Age), (2) a respiratory exchange ratio of >1.15 and (3) a plateau in *V*O_2_ indicated by an increase in *V*O_2_ of no more than 100 mL/min in the final two 20 sec periods of the test. Ventilatory threshold (VT) was determined using a combination of the v‐slope method (the point where a clear steeper increase in $\dot{V}{\mathrm{CO}}_{2}$ compared to $\dot{V}O_{2}$ occurs) and the ventilatory equivalent method (the point where $\dot{V}E/\dot{V}O_{2}$ rises without a concomitant rise in $\dot{V}E/\dot{V}{\mathrm{CO}}_{2}$). VT was assessed by 2 independent investigators with any differences between the two resolved by a third investigator when necessary (Gaskill et al. 2001).

#### Respiratory exchange ratio

Calculated from breath by breath measurements of CO_2_ and O_2_ concentrations taken during the *V*O_2_max test. To capture changes in RER across each percentile of the *V*O_2_max test, the area under the curve for was assessed for each subject for the full exercise duration.

#### Maximal voluntary muscle strength

Maximal voluntary strength (MVC) was assessed in the knee extensors of the dominant limb. Subjects were seated upright with their arms folded in a custom‐built dynamometer with their knee in 90° of flexion. Their lower leg (~3 cm proximal to the ankle) was strapped in a padded steel brace attached by a rigid bar to a strain gauge. Signals from the strain gauge were recorded on Spike 2 software via an analogue‐to‐digital converter (Cambridge Electronic Design (CED) 1401, UK) at a sampling rate of 2 kHz. Waist and shoulder straps were used to minimize any hip or upper body movement. The distance from the center of rotation of the knee joint to the steel brace was measured to allow torque to be calculated.

Subjects performed 3 MVCs during which maximal force as quickly as possible and hold the contraction for 3–4 sec. Strong verbal encouragement and visual feedback from a monitor placed in front of the subject were given. At least 1 min of rest was given between each contraction. The greatest MVC was used for analysis.

### Statistical analysis

The low subject numbers in this study mean that the emphasis of data analysis and interpretation is in the observed trends. However, statistical tests were still performed in order to gauge the degree of change from pre‐ to postexpedition. As this data set contains insufficient numbers for normality testing, a Gaussian distribution was assumed and differences in physiological variables pre‐ to postexpedition were identified using a paired Student T test. Statistical analysis and generation of graphs was conducted in Graphpad Prism.

## Results

### SpO_2,_ food intake, activity, and body weight data recorded during the expedition

SpO_2_ measures reported in response to a moderate intensity step test revealed a progressive decrease with increasing altitude, from 95.4 ± 1.5% (±SD) at 58 m to 89.4 ± 1.5% at 2752 m (Fig. 1C). This decrease was sustained, remaining at 89.2 ± 1.6 following 14 weeks at 2752 m and increasing to 92 ± 1.6% upon descent to 1700 m.

**Figure 2:**
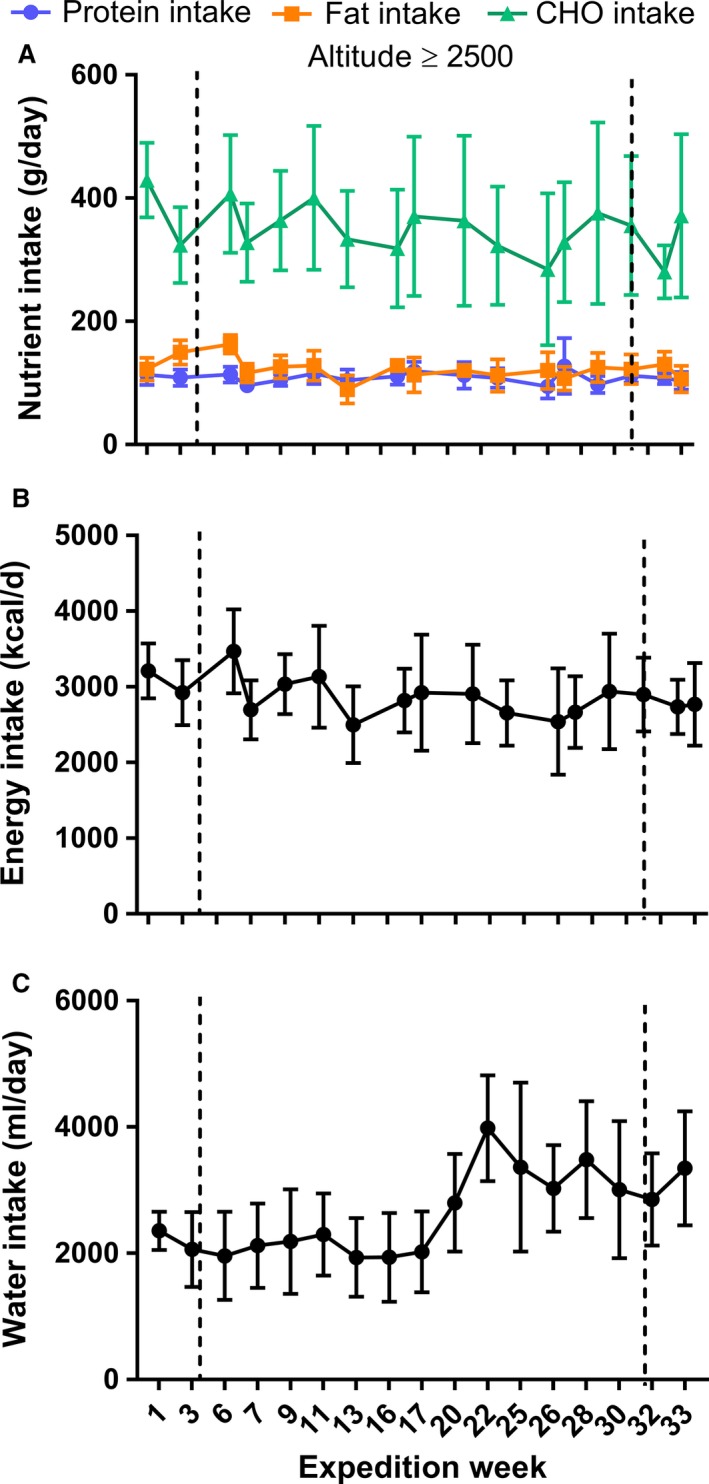
Nutrient (A), energy (B) and water (C) intake expressed as daily values throughout the winter expedition duration. Nutrient intake is broken down into protein, fat and carbohydrate (CHO) intake. Data were collected each day over the 7‐day period on the expedition week specified and is expressed as average + SD, *n* = 5.

The nutritional intake of the subjects, obtained from analysis of food diaries is detailed in Figure 2A. Total protein intake was 108.67 ± 18.17 g/day, carbohydrate intake 350 ± 101 g/day and fat intake 122 ± 25 g/day. The mean total energy intake was 2871.8 ± 543.1 kcal/day (Fig. 2B). Water intake was relatively constant (2167.0 ± 649.6 mL/day) between weeks 1 and 20 (Fig. 2C), after which it increased as did the inter‐individual differences, the latter reflected by the increasing SD (3294.2 ± 934.0 mL/day).

**Figure 3:**
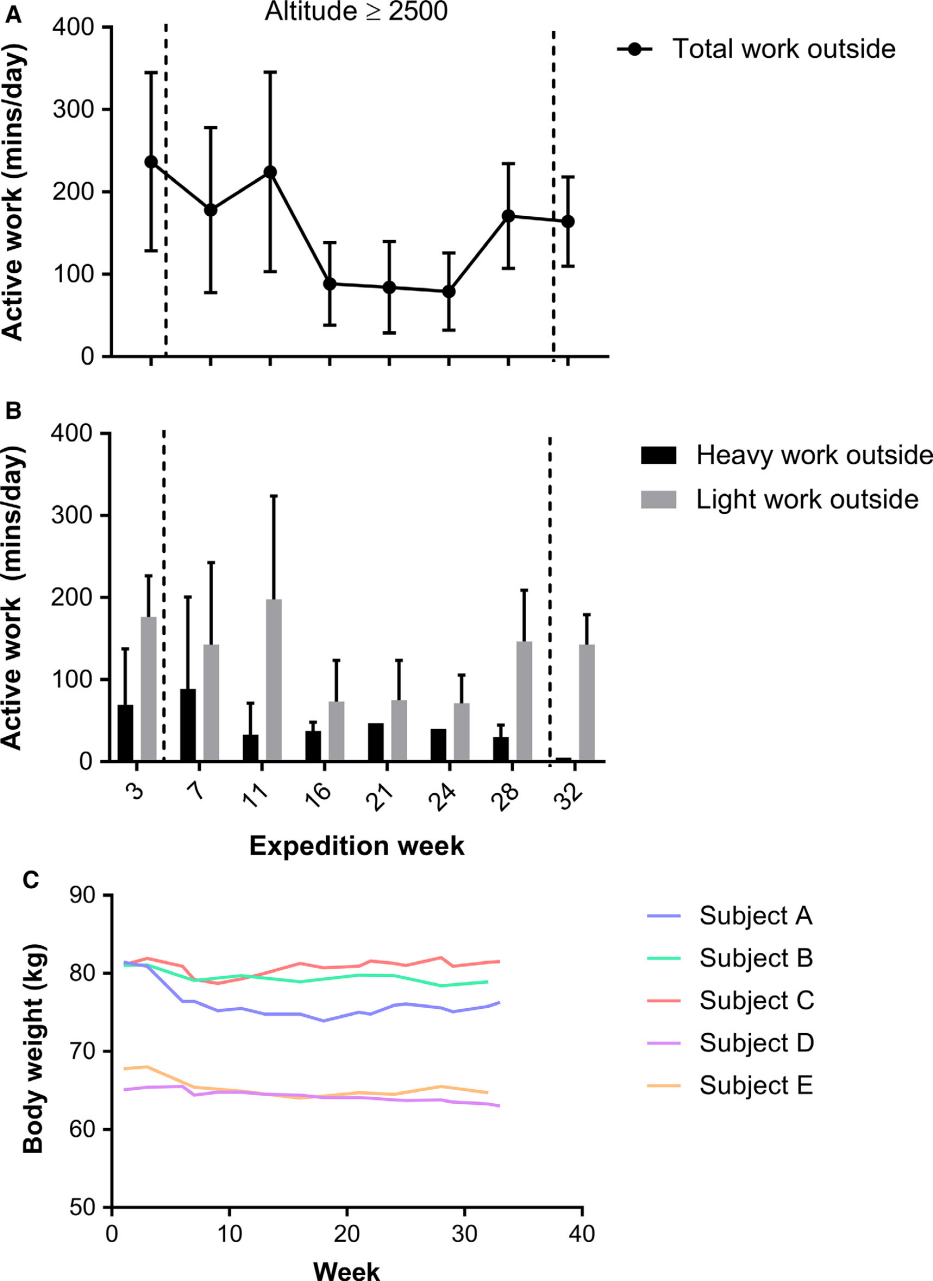
Work performed outside and body weight recorded during the 33 week attempted Antarctica winter crossing. Depicted as total work performed (A) and as an estimate of the division between heavy and light work (B) and body weight (C). Work data were collected each day over the 7‐day period on the expedition week specified and is expressed as average of this +SD, *n* = 5.

Outdoor exposure varied throughout the expedition and between subjects. Total time spent outside averaged 153 ± 94 mins/day (Fig. 3A) with light and heavy work (Fig. 3B) accounting for 128 ± 80 and 44 ± 48 min/day, respectively. A drop in average activity levels can be observed following week 11, coinciding with the point at which winter camp was established, before increasing once more upon descent from winter camp (week 28). Light work was performed indoors and remained constant at 60 min/day for each subject throughout the duration of the expedition.

Body weight data obtained during the expedition revealed an average decrease in 2.56 ± 2.15 kg from week 1 to week 32 (Fig. 3C). Statistically, this difference was close to significance (*P* = 0.063).

### Pre: postexpedition measurements

#### Serum metabolic profile changes

**Figure 4:**
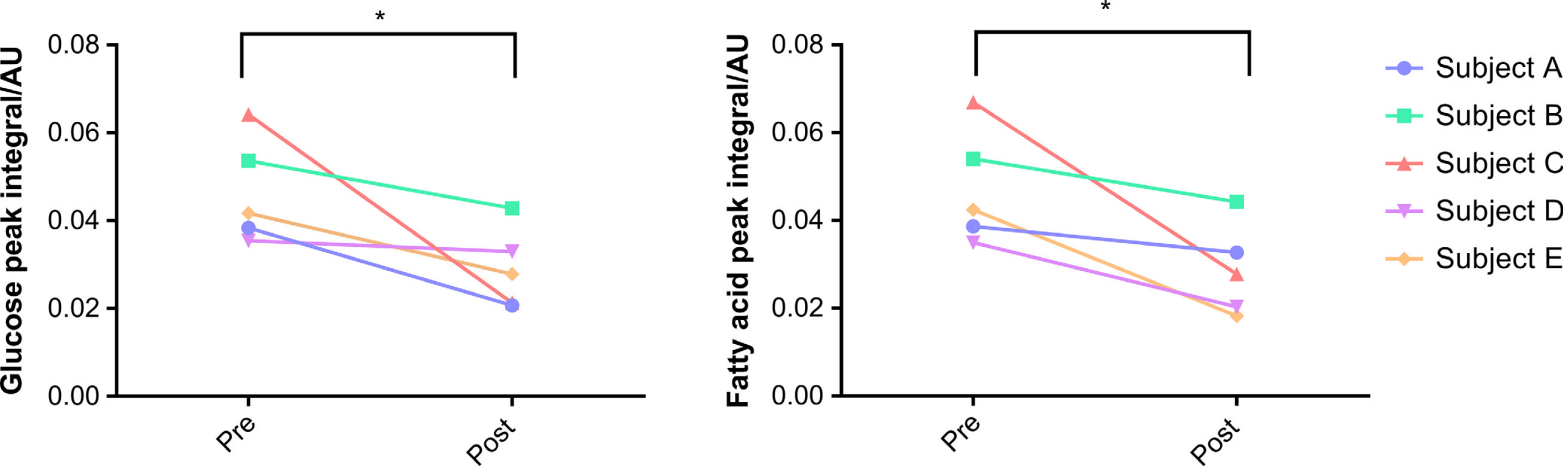
Serum metabolic profile analysis. Peak integrals undergoing significant decreases within principal component 2. This includes the peaks of glucose and the shoulder of the fatty acid CH2 resonance, displayed as arbitrary units (AU), * *P* ≤ 0.05.

Multivariate statistics revealed a reduction in principal component 2 (PC2) postexpedition, which captured ~30% of variance in the metabolic profiling data (Fig. S1A). The separation of variables by PC2 is demonstrated in (Fig. S1B). Within PC2, peaks corresponding to glucose and a fatty acid CH_2_ resonance underwent the most prominent alterations, decreasing by 35.9 (±22.9)% and 39.6 (±20.6)% respectively (*P* < 0.05) (Fig. 4). Unfortunately, it was not possible to identify the subtype of fatty acid from which this resonance was derived.

#### Anthropometric and functional physiological measurements

**Figure 5:**
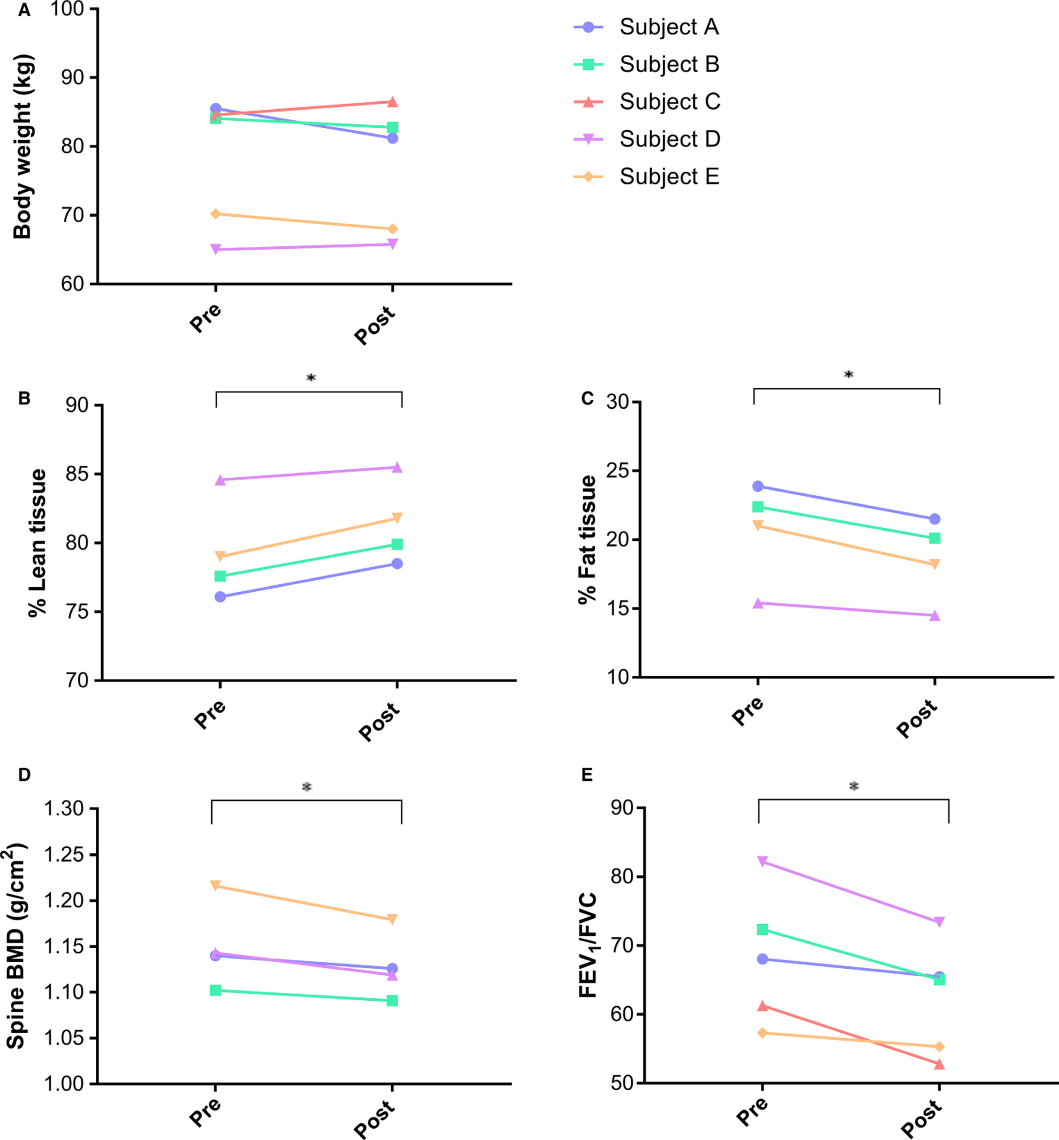
Anthropometric and physiological measurements taken pre‐ and postexpedition. Body weight (kg) (A), % lean tissue (B), % fat tissue (C), spine BMD (bone mineral density) (D), the ratio of forced expiratory volume in 1 second (FEV1)/forced vital capacity (FVC) (E). Data presented as individual subject values, * *P* ≤ 0.05, *n* = 4–5.

The anthropometric and physiological measurements taken pre‐ and postexpedition and are summarized in Table 1. No significant changes in body weight (Fig. 5A) or BMI (pre = 26.4 ± 3.9 vs. post = 26.0 ± 3.5 kg/m^2^) were observed postexpedition. However, an increase in % lean and a decrease in % fat tissue, by 2.1 (±0.8) % (*P* < 0.05) were observed postexpedition (Fig. 5B and C). This change was also reflected through a 2.0 (±1.1 kg) decrease in body fat (*P* ˂ 0.05). In addition, a 1.8 (±0.9) % decrease (*P* < 0.05) was observed in spine BMD (Fig. 5D).

**Table 1:** Summary of changes in anthropometric variables, resting lung function and cardiovascular parameters pre‐ and postexpedition

Variable	Mean pre (±SD)	Mean post (±SD)	Difference	*P* value
% Lean tissue	79.3 (3.7)	81.4 (3.0)	2.1 (0.8)	0.015^a^
% Fat tissue	20.7 (3.7)	18.6 (3.0)	−2.1 (0.8)	0.015^a^
Body fat (kg)	16.0 (4.7)	14.0 (3.7)	−2.0 (1.1)	0.034^a^
Body weight (kg)	77.9 (9.58)	76.9 (9.3)	−1.02 (2.5)	0.41
Spine BMD g/cm^2^	1.2 (0.05)	1.13 (0.04)	−0.02 (0.01)	0.035^a^
FVC (L)	6.38 (1.29)	7.11 (1.88)	0.72 (0.62)	0.060
FEV1	4.29 (0.65)	4.34 (0.71)	0.05 (0.22)	0.661
FEV_1_/ FVC%	68.2 (9.7)	62.4 (8.4)	−5.8 (3.3)	0.016^a^
Heart rate (bpm)	60 (6)	63 (7)	3 (7)	0.39
sBP	123 (6)	121 (5)	−2 (8)	0.61
dBP	72 (8)	75 (9)	2 (6)	0.37
MAP	93 (9)	93 (7)	−0.04 (5.7)	0.99

BMD, bone mineral density; FVC, forced vital capacity; FEV_1_,forced expiratory volume in 1 sec, bpm, beats per minute, sBP , systolic blood pressure, dBP, diastolic blood pressure, MAP , mean arterial blood pressure.

*n* = 4–5, values presented as mean ± SD.

a Denotes significance, *P* < 0.05.

Lung function measurements revealed no significant change in either FEV_1_ (L) or FVC (L). However, FVC numerically increased in all subjects postexpedition, a change that was close to significant (*P* = 0.06) and that is reflected in the decrease in the FEV_1_/FVC% by 5.8 (±3.3) % postexpedition (*P* < 0.05) (Fig 5E).

Regarding cardiovascular measures, no significant changes were observed in resting blood pressure (sBP, dBP or MAP) or heart rate.

#### Exercise data

**Figure 6:**
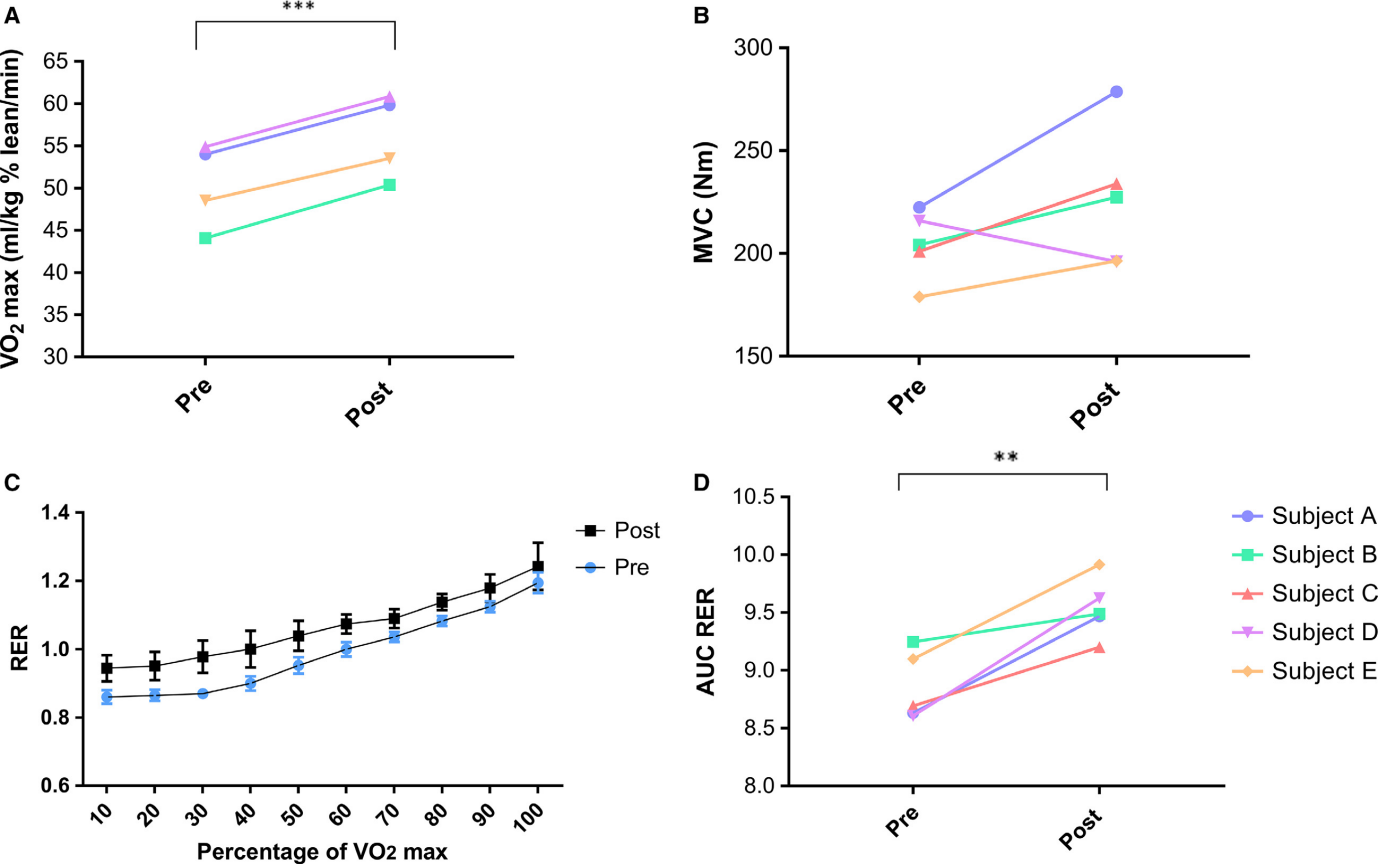
Exercise parameters measured pre‐ and postexpedition. *V*O_2_ max corrected to % lean mass (A), Maximal voluntary contraction (MVC) (B), average RER (+SD) presented at each 10th percentile of a *V*O_2_ max test (C), area under the curve (AUC) for RER (D). Data presented as individual subject values, **P < 0.01, ***P < 0.001, *n* = 4–5.

Physiological measures related to exercise are displayed in Table 2. An 11.6 (±1.9) % increase (*P* > 0.001) was observed in *V*O_2_max corrected to lean mass postexpedition (Fig 6A). A 7.8 (±3.6) % increase (*P* > 0.01) in AUC for RER was observed postexpedition compared to pre (*P* < 0.01). The average (mean) RER of the group through each percentile of the *V*O_2_max test is depicted in Fig 6C, with individual differences displayed in Fig 6D.

**Table 2:** Summary of changes in exercise parameters pre‐ and postexpedition

Variable	Mean pre (±SD)	Mean post (±SD)	Difference (±SD)	*P* value
*V*O_2_ max (L/min)	3.2 (0.6)	3.5 (0.4)	0.2 (0.3)	0.163
*V*O_2_ max (mL/kg/min)	42.1 (4.9)	45.5 (3.8)	3.38 (5.3)	0.187
*V*O_2_ max (mL/kg lean tissue)	50.4 (5.1)	56.1 (5.0)	5.8 (0.6)	0.0003^b^
Max heart rate (bpm)	191.6 (5.6)	189.6 (5.1)	−2.0 (6.8)	0.546
AUC RER	8.85 (0.30)	9.54 (0.26)	0.68 (0.31)	0.007^a^
MVC (Nm)	204.5 (16.7)	226.5 (33.9)	22.0 (27.7)	0.062

*V*O_2_, pulmonary oxygen uptake; bpm‐beats per minute; bpm‐beats per minute RER, respiratory exchange ratio; AUC, area under the curve, MVC, maximal voluntary contraction; Nm, Newton meters. *n* = 4–5, values presented as mean ± SD.

a Denotes significance, *P* < 0.01,

b Denotes significance *P* < 0.001.

Examination of maximal muscle strength in the dominant leg revealed an increase in all subjects bar one postexpedition (Fig 6B). The trend towards an increase was reflected in the *P* value that was close to significance (*P* = 0.06).

## Discussion

The Antarctic winter is amongst the most extreme environments on earth and so presents significant physiological challenges. Up on the Antarctic plateau, high altitudes (and so hypobaric hypoxia) are combined with monthly mean temperatures falling below −60°C, strong magnetic fields, high UV radiation and wind velocities as well as altered dark/light cycles and psychological stressors. In this small applied field study, the use of protective clothing and cabin shelter meant exposure to the majority of these environmental extremes beyond peripheries was kept to a minimum. In addition, nutritional intake remained within recommended daily levels (Trumbo et al. 2002) and a moderate level of physical activity was maintained. Changes observed in the array of physiological parameters and metabolic profile measures obtained pre‐ and postexpedition are therefore most likely a result of acclimatization to chronic hypobaric hypoxia as opposed to other perturbations. The stress of this moderate hypoxic exposure is apparent in the progressive decrease in SpO_2_ reported with increasing altitude, an effect sustained throughout the expedition.

Amongst these physiological changes were a number of measures that were suggestive of metabolic remodeling. This includes anthropometric measures revealing a loss of body fat and assessment of resting serum metabolic profiles, which highlighted changes in glucose and fatty acid resonances. In relation to physical exertion, these changes were accompanied by an increase in RER, thus indicating an increased reliance upon carbohydrate utilization during incremental exercise.

The exact causes or consequences of these shifts are unclear. Alterations in carbohydrate and fat metabolism have both been identified as having key roles in the response to short term hypobaric hypoxia in skeletal muscle (Horscroft and Murray 2014). It is therefore possible that changes in substrate utilization are occurring in response to chronic hypoxia at a whole‐body level and that this is reflected both in anthropometric measures and substrate utilization during exercise. Indeed, the increase in RER is in line with previous reports of humans undergoing exercise in hypoxia following a prolonged (40 day) exposure (Sutton et al. 1988). However, comparison to the present study is limited given the differences in duration and severity of exposure and the fact that the measures of this study were obtained at sea level. The in‐depth investigation into metabolic function that included use of metabolomics did not, therefore, highlight novel alterations. Instead, the changes identified do suggest that the effects of chronic hypobaric hypoxic exposure are in line with those reported in previous shorter term investigations.

In relation to body composition changes, the proportionate increase in % lean mass and decrease in fat mass may be indicative of alterations to lipid storage capacity as hypoxic exposure has been associated with fat store mobilization (Young et al. 1989; Wood et al. 2011; Suzuki et al. 2014). This concept would align well with the shifts away from fat towards carbohydrate utilization, as indicated by the RER results of this study.

Previous high altitude studies reveal mixed results regarding body composition changes, with reports of an initial loss of fat mass followed by lean and vice versa (Boyer and Blume 1984; Guilland and Klepping 1985; Rose et al. 1988). Interestingly, these prior studies tend to observe that changes in body composition are accompanied by a loss of body weight, the degree of which increases with increasing altitude (Boyer and Blume 1984; Guilland and Klepping 1985; Rose et al. 1988). For instance, Caucasian subjects exposed to 5400 m demonstrated a 1.5–1.9 kg loss in body weight, which increased to 3.4–4 kg at 7100 m (Boyer and Blume 1984; Guilland and Klepping 1985). However, weight loss at altitude is not reported across all studies, with subjects being exposed to 3,454 m for 28 days reporting no change in either body weight or fat mass (Jacobs et al. 2012). This suggests that maintaining body weight becomes an issue at more extreme altitudes (above ~3500 m) and may result from the commonly reported loss of appetite (Vats et al. 2004). The lack of body weight change in subjects of the present study, both during the expedition and measured pre and post, may therefore be due to the altitude exposure being at the lower end of the spectrum (~2500 m). The consistent nutrient intake suggests loss of appetite was not experienced. The lack of body weight change may also be reflective of the prolonged nature of the study, with body weight stabilizing over the duration of the expedition as subjects acclimatized.

At a functional level, the apparent shifts in metabolic signature and body composition were accompanied by significant improvements in *V*O_2_max corrected to % lean tissue. Although not significant, trends were also observed for increasing MVC and FVC in most subjects postexpedition, the latter contributing to the significant increase in FEV_1_/FVC. Together, these changes could be a reflection of a training effect. However, this is unlikely given that the majority of heavy physical exertion was undertaken during the first 11 weeks, after which levels declined. It is also not possible to draw such conclusions given that there is an absence of baseline activity levels nor accurate measures of exercise severity. In particular, the improvements in *V*O_2_max could reflect potential benefits of hypoxic acclimatization upon O_2_ delivery, for instance resulting from increasing erythropoiesis and angiogenesis (Shweiki et al. 1992; Rodriguez et al. 1999).

Given previous reports of both cold and high altitude exposure having detrimental effects upon lung function (Welsh et al. 1993; Cotes et al. 2006; Ziaee et al. 2008), it may perhaps appear surprising that FVC tended towards an increase. However, the translation of this prior work to this study is limited given differences in the degree and severity of altitude exposure and the fact that spirometry measures of this study were conducted at sea level, meaning altitude induced obstruction is not relevant.

A novel measure amongst high altitude studies is the decrease in spine BMD. Whilst slight, the decline reported here is in line with that associated with increasing fracture risk (Nguyen et al. 1993). Whilst the precise etiology of this is not clear, it may be related to the effect of hypoxia upon the regulation of bone density (Arnett et al. 2003; Utting et al. 2006) or HIF‐1α regulation on osteoclasts (Knowles 2015). Other factors known to impact BMD include decreases in activity levels (Mazzeo et al. 1998; Vuori 2001), although the maintenance of activity levels throughout and reported improvement in functional measures suggest this is unlikely to be the case. It could also relate to the altered day‐night cycle experienced during the Antarctic winter in turn impacting altering vitamin D intake. Sunlight exposure is required to satisfy vitamin D requirements (Holick 2004), therefore, the prolonged lack of sunlight exposure resulted in subjects having insufficient vitamin D levels. Similarly dietary changes in Ca^2+^ (Kelly et al. 1990), vitamin C (Aghajanian et al. 2015) and D (Bischoff‐Ferrari et al. 2004) can also influence BMD. However, as details of Ca^2+^, vitamin C and D intake were not recorded, it is not possible to draw any meaningful conclusion.

### Study limitations

This study presents a unique and novel exploration of the effects of an extreme environment upon human physiology. However, as the main aim of the expedition was to cross Antarctica as opposed to conducting a robust scientific experiment, there are a number of limitations in the study design and subsequent data interpretation.

Firstly, the small subject numbers severely limits statistical power and there was no control group. Secondly, for logistical reasons, the majority of physiological measures were taken 6 weeks prior to and 4 days postexpedition. This is particularly limiting for those measures prone to rapid fluxes, including metabolic profile and to a lesser extent body composition and lung function. This is less of a concern for measures of exercise performance given that previous studies have demonstrated maintenance of muscle energetics in response to altitude exposure 1 week postexposure (Edwards et al. 2010).

## Conclusion

We have presented the first in‐depth examination of human physiology and metabolism in response to prolonged exposure to high altitude (~2500 m) in the Antarctic winter. It has highlighted key areas of interest for future investigations, particularly in the context of chronic hypoxic exposure and prolonged expeditions of a similar nature. This includes a change in metabolic signature, involving alterations to both glucose and fatty acid homeostasis with a shift towards increased reliance on carbohydrate metabolism during exercise and a reduction in body fat. This was accompanied by an improvement in *V*O_2_max. In addition, spine BMD and lung function measures were identified as novel parameters of interest.

## Conflict of Interests

None declared.

## Data Accessibility

## Supporting information




**Figure S1.** Principal component analysis of serum metabolic profile. Scree plot (A) demonstrating the variance explained by each principal component. Scores plot of principal components 1 and 2 where green corresponds to pre expedition and red post (B).

## References

[phy213613-bib-0001] Aghajanian, P. , S. Hall , M. D. Wongworawat , and S. Mohan . 2015 The roles and mechanisms of actions of vitamin C in bone: new developments. J. Bone Miner. Res. 30:1945–1955. 26358868 10.1002/jbmr.2709 PMC4833003

[phy213613-bib-0002] Arnett, T. R. , D. C. Gibbons , J. C. Utting , I. R. Orriss , A. Hoebertz , M. Rosendaal , et al. 2003 Hypoxia is a major stimulator of osteoclast formation and bone resorption. J. Cell. Physiol. 196:2–8. 12767036 10.1002/jcp.10321

[phy213613-bib-0003] Behrooz, A. , and F. Ismail‐Beigi . 1999 Stimulation of glucose transport by hypoxia: signals and mechanisms. Physiology 14:105–110. 10.1152/physiologyonline.1999.14.3.105 11390832

[phy213613-bib-0004] Bischoff‐Ferrari, H. A. , T. Dietrich , E. J. Orav , and B. Dawson‐Hughes . 2004 Positive association between 25‐hydroxy vitamin D levels and bone mineral density: a population‐based study of younger and older adults. Am. J. Med. 116:634–639. 15093761 10.1016/j.amjmed.2003.12.029

[phy213613-bib-0005] Blatteis, C. , and L. Lutherer . 1976 Effect of altitude exposure on thermoregulatory response of man to cold. J. Appl. Physiol. 41:848–858. 1002639 10.1152/jappl.1976.41.6.848

[phy213613-bib-0006] Boyer, S. J. , and F. D. Blume . 1984 Weight loss and changes in body composition at high altitude. J. Appl. Physiol. 57:1580–1585. 6520055 10.1152/jappl.1984.57.5.1580

[phy213613-bib-0007] Cotes, J. E. , D. J. Chinn , and M. R. Miller . 2006 Lung function: physiology, measurement and application in medicine. 6th ed. Blackwell Scientific Pulications, Oxford, U.K.

[phy213613-bib-0008] Curtis, K. J. , K. A. O'Brien , R. J. Tanner , J. I. Polkey , M. Minnion , M. Feelisch , et al. 2015 Acute dietary nitrate supplementation and exercise performance in COPD: a double‐blind, placebo‐controlled, randomised controlled pilot study. PLoS ONE 10:e0144504. 26698120 10.1371/journal.pone.0144504 PMC4689520

[phy213613-bib-0009] De Meyer, T. , D. Sinnaeve , B. Van Gasse , E. Tsiporkova , E. R. Rietzschel , M. L. De Buyzere , et al. 2008 NMR‐based characterization of metabolic alterations in hypertension using an adaptive, intelligent binning algorithm. Anal. Chem. 80:3783–3790. 18419139 10.1021/ac7025964

[phy213613-bib-0010] Dieterle, F. , A. Ross , G. Schlotterbeck , and H. Senn . 2006 Probabilistic quotient normalization as robust method to account for dilution of complex biological mixtures. Application in 1H NMR metabonomics. Anal. Chem. 78:4281–4290. 16808434 10.1021/ac051632c

[phy213613-bib-0011] Edwards, L. M. , A. J. Murray , D. J. Tyler , G. J. Kemp , C. J. Holloway , P. A. Robbins , et al. 2010 The effect of high‐altitude on human skeletal muscle energetics: 31P‐MRS results from the caudwell xtreme everest expedition. PLoS ONE 5:e10681. 20502713 10.1371/journal.pone.0010681 PMC2873292

[phy213613-bib-0012] Gaskill, S. E. , B. C. Ruby , A. J. Walker , O. A. Sanchez , R. C. Serfass , and A. S. Leon . 2001 Validity and reliability of combining three methods to determine ventilatory threshold. Med. Sci. Sports Exerc. 33:1841–1848. 11689733 10.1097/00005768-200111000-00007

[phy213613-bib-0013] Guilland, J. , and J. Klepping . 1985 Nutritional alterations at high altitude in man. Eur. J. Appl. Physiol. 54:517–523. 10.1007/BF00422963 4085482

[phy213613-bib-0014] Hochachka, P. W. , C. L. Beatty , Y. Burelle , M. E. Trump , D. C. McKenzie , and G. O. Matheson . 2002 The lactate paradox in human high‐altitude physiological performance. Physiology 17:122–126. 10.1152/nips.01382.2001 12021383

[phy213613-bib-0015] Holick, M. F. 2004 Sunlight and vitamin D for bone health and prevention of autoimmune diseases, cancers, and cardiovascular disease. Am. J. Clin. Nutr. 80:1678S–1688S. 15585788 10.1093/ajcn/80.6.1678S

[phy213613-bib-0016] Horscroft, J. A. , and A. J. Murray . 2014 Skeletal muscle energy metabolism in environmental hypoxia: climbing towards consensus. Extrem. Physiol. Med. 3:19. 25473486 10.1186/2046-7648-3-19 PMC4253994

[phy213613-bib-0017] Jacobs, R. A. , C. Siebenmann , M. Hug , M. Toigo , A.‐K. Meinild , and C. Lundby . 2012 Twenty‐eight days at 3454‐m altitude diminishes respiratory capacity but enhances efficiency in human skeletal muscle mitochondria. FASEB J. 26:5192–5200. 22968913 10.1096/fj.12-218206

[phy213613-bib-0018] Kelly, P. J. , N. A. Pocock , P. N. Sambrook , and J. A. Eisman . 1990 Dietary calcium, sex hormones, and bone mineral density in men. BMJ 300:1361–1364. 2142609 10.1136/bmj.300.6736.1361 PMC1662977

[phy213613-bib-0019] Knowles, H. J. 2015 Hypoxic regulation of osteoclast differentiation and bone resorption activity. Hypoxia 3:73. 27774484 10.2147/HP.S95960 PMC5045091

[phy213613-bib-0020] López‐Barneo, J. , R. Pardal , and P. Ortega‐Sáenz . 2001 Cellular mechanism of oxygen sensing. Annu. Rev. Physiol. 63:259–287. 11181957 10.1146/annurev.physiol.63.1.259

[phy213613-bib-0021] Mazzeo, R. S. , P. Cavanagh , W. J. Evans , M. Fiatarone , J. Hagberg , E. McAuley , et al. 1998 Exercise and physical activity for older adults. Med. Sci. Sports Exerc. 30:992–1008. 9624662

[phy213613-bib-0500] Miller, M. R. , J. Hankinson , V. Brusasco , F. Burgos , R. Casaburi , A. Coates , et al. 2005 Standardisation of spirometry. Eur. Respir. J. 26:319–338. 16055882 10.1183/09031936.05.00034805

[phy213613-bib-0022] Nguyen, T. , P. Sambrook , P. Kelly , G. Jones , S. Lord , J. Freund , et al. 1993 Prediction of osteoporotic fractures by postural instability and bone density. BMJ 307:1111–1115. 8251809 10.1136/bmj.307.6912.1111 PMC1679116

[phy213613-bib-0023] Robinson, K. A. , and E. M. Haymes . 1990 Metabolic effects of exposure to hypoxia plus cold at rest and during exercise in humans. J. Appl. Physiol. 68:720–725. 2390141 10.1152/jappl.1990.68.2.720

[phy213613-bib-0024] Rodriguez, F. A. , H. Casas , M. Casas , T. PagÉs , R. Rama , A. Ricart , et al. 1999 Intermittent hypobaric hypoxia stimulates erythropoiesis and improves aerobic capacity. Med. Sci. Sports Exerc. 31:264–268. 10063816 10.1097/00005768-199902000-00010

[phy213613-bib-0025] Rose, M. S. , C. S. Houston , C. S. Fulco , G. Coates , J. R. Sutton , and A. Cymerman . 1988 Operation Everest. II: Nutrition and body composition. J. Appl. Physiol. 65:2545–2551. 3215854 10.1152/jappl.1988.65.6.2545

[phy213613-bib-0026] Semenza, G. L. 1999 Regulation of mammalian O_2_ homeostasis by hypoxia‐inducible factor 1. Annu. Rev. Cell Dev. Biol. 15:551–578. 10611972 10.1146/annurev.cellbio.15.1.551

[phy213613-bib-0027] Semenza, G. L. , P. H. Roth , H.‐M. Fang , and G. L. Wang . 1994 Transcriptional regulation of genes encoding glycolytic enzymes by hypoxia‐inducible factor 1. J. Biol. Chem. 269:23757–23763. 8089148

[phy213613-bib-0028] Shweiki, D. , A. Itin , D. Soffer , and E. Keshet . 1992 Vascular endothelial growth factor induced by hypoxia may mediate hypoxia‐initiated angiogenesis. Nature 359:843. 1279431 10.1038/359843a0

[phy213613-bib-0029] Siebenmann, C. , P. Rasmussen , M. Hug , S. Keiser , D. Flück , J. P. Fisher , et al. 2016 Parasympathetic withdrawal increases heart rate after two weeks at 3454 m altitude. The Journal of Physiology 595:1619–1626. 10.1113/JP273726 PMC5330924 27966225

[phy213613-bib-0030] Siervo, M. , H. L. Riley , B. O. Fernandez , C. A. Leckstrom , D. S. Martin , K. Mitchell , et al. 2014 Effects of prolonged exposure to hypobaric hypoxia on oxidative stress, inflammation and gluco‐insular regulation: the not‐so‐sweet price for good regulation. PLoS ONE 9:e94915. 24733551 10.1371/journal.pone.0094915 PMC3986261

[phy213613-bib-0031] Sutton, J. R. , J. T. Reeves , P. D. Wagner , B. M. Groves , A. Cymerman , M. K. Malconian , et al. 1988 Operation Everest II: oxygen transport during exercise at extreme simulated altitude. J. Appl. Physiol. 64:1309–1321. 3132445 10.1152/jappl.1988.64.4.1309

[phy213613-bib-0032] Suzuki, T. , S. Shinjo , T. Arai , M. Kanai , and N. Goda . 2014 Hypoxia and fatty liver. World J. Gastroenterol. 20:15087. 25386057 10.3748/wjg.v20.i41.15087 PMC4223242

[phy213613-bib-0033] Trumbo, P. , S. Schlicker , A. A. Yates , and M. Poos . 2002 Dietary reference intakes for energy, carbohydrate, fiber, fat, fatty acids, cholesterol, protein and amino acids. J. Am. Diet. Assoc. 102:1621–1630. 12449285 10.1016/s0002-8223(02)90346-9

[phy213613-bib-0034] Utting, J. , S. Robins , A. Brandao‐Burch , I. Orriss , J. Behar , and T. Arnett . 2006 8Hypoxia inhibits the growth, differentiation and bone‐forming capacity of rat osteoblasts. Exp. Cell Res. 312:1693–1702. 16529738 10.1016/j.yexcr.2006.02.007

[phy213613-bib-0035] Vallerand, A. L. , and I. Jacobs . 1989 Rates of energy substrates utilization during human cold exposure. Eur. J. Appl. Physiol. 58:873–878. 10.1007/BF02332221 2767069

[phy213613-bib-0036] Vats, P. , S. N. Singh , R. Shyam , V. K. Singh , S. B. Singh , P. K. Banerjee , et al. 2004 Leptin may not be responsible for high altitude anorexia. High Alt. Med. Biol. 5:90–92. 15072723 10.1089/152702904322963753

[phy213613-bib-0037] Vuori, I. M. 2001 Dose‐response of physical activity and low back pain, osteoarthritis, and osteoporosis. Med. Sci. Sports Exerc. 33(6 Suppl):S551–S586; discussion 609‐510. 11427782 10.1097/00005768-200106001-00026

[phy213613-bib-0038] Welsh, C. H. , P. D. Wagner , J. T. Reeves , D. Lynch , T. M. Cink , J. Armstrong , et al. 1993 Operation Everest II: spirometric and radiographic changes in acclimatized humans at simulated high altitudes. Am. Rev. Respir. Dis. 147:1239. 8484637 10.1164/ajrccm/147.5.1239

[phy213613-bib-0039] Wood, I. S. , T. Stezhka , and P. Trayhurn . 2011 Modulation of adipokine production, glucose uptake and lactate release in human adipocytes by small changes in oxygen tension. Pflügers Archiv 462:469. 21698390 10.1007/s00424-011-0985-7

[phy213613-bib-0040] Woolcott, O. O. , M. Ader , and R. N. Bergman . 2015 Glucose homeostasis during short‐term and prolonged exposure to high altitudes. Endocr. Rev. 36:149–173. 25675133 10.1210/er.2014-1063 PMC4399271

[phy213613-bib-0041] Young, P. M. , M. S. Rose , J. R. Sutton , H. J. Green , A. Cymerman , and C. S. Houston . 1989 Operation Everest II: plasma lipid and hormonal responses during a simulated ascent of Mt Everest. J. Appl. Physiol. 66:1430–1435. 2651390 10.1152/jappl.1989.66.3.1430

[phy213613-bib-0042] Ziaee, V. , R. Alizadeh , and A. Movafegh . 2008 Pulmonary function parameters changes at different altitudes in healthy athletes. Iranian Journal of Allergy, Asthma and Immunology 7:79–84. 18552409

