# Supplementary figures and images for: Human physiological and metabolic responses to an attempted winter crossing of Antarctica: the effects of prolonged hypobaric hypoxia
Source: Physiol Rep. 2018 Mar 9;6(5):e13613. doi: 10.14814/phy2.13613 (PMC5843758; doi:10.14814/phy2.13613)

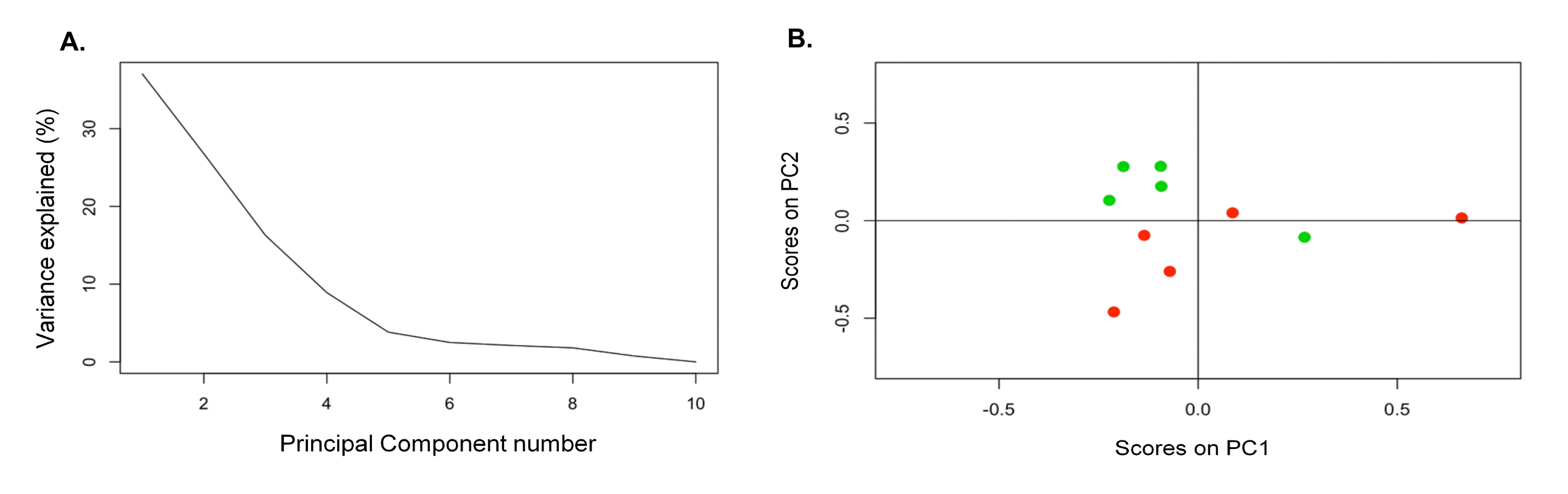

Supplement: Supplementary file 1 — Figure S1. Principal component analysis of serum metabolic profile. Scree plot (A) demonstrating the variance explained by each principal component. Scores plot of principal components 1 and 2 where green corresponds to pre expedition and red post (B). [file PHY2-6-e13613-s001.tif]
